# Case Report: Reversible Hyperglycemia Following Rapamycin Treatment for Atypical Choroid Plexus Papilloma in an Infant

**DOI:** 10.3389/fendo.2022.865913

**Published:** 2022-07-05

**Authors:** Jiale Liu, Minjie Luo, Siyuan Lv, Shaohua Tao, Zhu Wu, Lihua Yu, Danna Lin, Lulu Huang, Li Wu, Xu Liao, Juan Zi, Xiaorong Lai, Yuting Yuan, Wangming Zhang, Lihua Yang

**Affiliations:** ^1^ Department of Pediatric Hematology, Zhujiang Hospital, Southern Medical University, Guangzhou, China; ^2^ Department of Pediatric Neurosurgery, Zhujiang Hospital, Southern Medical University, Guangzhou, China; ^3^ Department of Pediatric Intensive Care Unit, Zhujiang Hospital, Southern Medical University, Guangzhou, China

**Keywords:** rapamycin, reversible hyperglycemia, insulin, atypical choroid plexus papilloma, infant, case report

## Abstract

In this study, atypical choroid plexus papilloma was treated with high-dose rapamycin for 17 days preoperatively in an infant. Rapamycin significantly reduced the blood supply to the tumor while reducing the tumor volume, and most of the tumor was resected successfully. However, the infant developed hyperglycemia related to the rapamycin dose, which was effectively controlled by adjusting the dose and applying insulin.

## Introduction

Atypical choroid plexus papilloma (aCPP), a subtype of a choroid plexus tumor (CPT) (1–3), is a rare central nervous system tumor characterized by an abundant blood supply and the risk of massive blood loss.

Rapamycin has been widely applied to prevent acute rejection in organ transplantation and successfully treats complicated vascular anomalies and some brain tumors (4, 5).

Here, we report an 11-month-old infant with aCPP treated with high-dose rapamycin for 17 days preoperatively. Rapamycin is effective in reducing the blood supply to the tumor, controlling tumor volume and reducing risk of surgical hemorrhage. However, the infant developed hyperglycemia related to the rapamycin dose, which was effectively controlled by adjusting the dose and applying insulin.

## Case Presentation

An 11-month-old boy (weight 10 kg) was admitted to the pediatric intensive care unit (PICU) on May 4, 2021 because of increasing head circumference for 1 week and a cerebral hernia for 1 day. He presented with irritability, dysphoria, and vomiting, an enlarged head circumference of 50 cm, and an approximate 3*3 cm anterior fonticulus with increasing tension. He was somnolent with obvious nuchal rigidity but had normal pupils, isometric muscle tension, and normal spinal reflexes at admission. He had a negative family history of neurologic or metabolic diseases.

Routine blood examination, blood biochemistry assays, functional coagulation assays, endocrine function, and diabetes mellitus–associated laboratory tests were normal, except for a slight increase in cortisol levels at 577 nmol/L (May 4, 2021, 23:30) (reference value 166–507 nmol/L at 8:00; 73.8-291 nmol/L at 16:00), which returned to normal 1 week later and might be related to the infant’s state of increased stress. Computed tomography (CT) and magnetic resonance (MR) images revealed a primary malignant tumor (60.00*84.01*94.72 mm) with a rich blood supply in the right lateral ventricle and metastatic tumors in the left lateral ventricle, the third ventricle, and cerebellopontine angle (CPA) (**Figure 2**).

**Figure 1:**
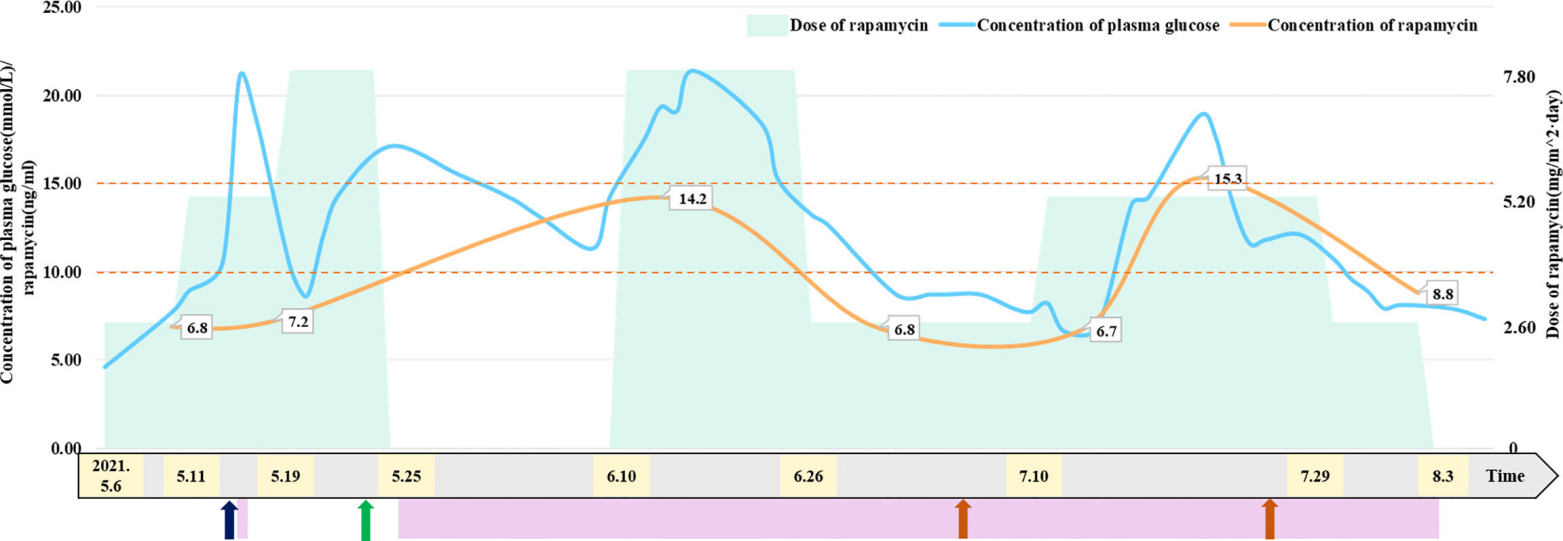
Relationship between dose of rapamycin and concentration of plasma glucose and rapamycin. Patient received insulin treatment to control plasma glucose on May 15 and from May 25 to August 3, 2021. Patient underwent Ommaya insertion and biopsy on May 13, 2021. Patient underwent subtotal resection of tumor on May 23, 2021. Patient received CPT-SIOP-2000 chemotherapy on July 2 and July 26, 2021. The maintaining trough concentration of rapamycin is recommended between 10 to 15 ng/mL. The figure indicated that patient’s concentration of plasma glucose has a positive correlation with the dose of rapamycin.

**Figure 2:**
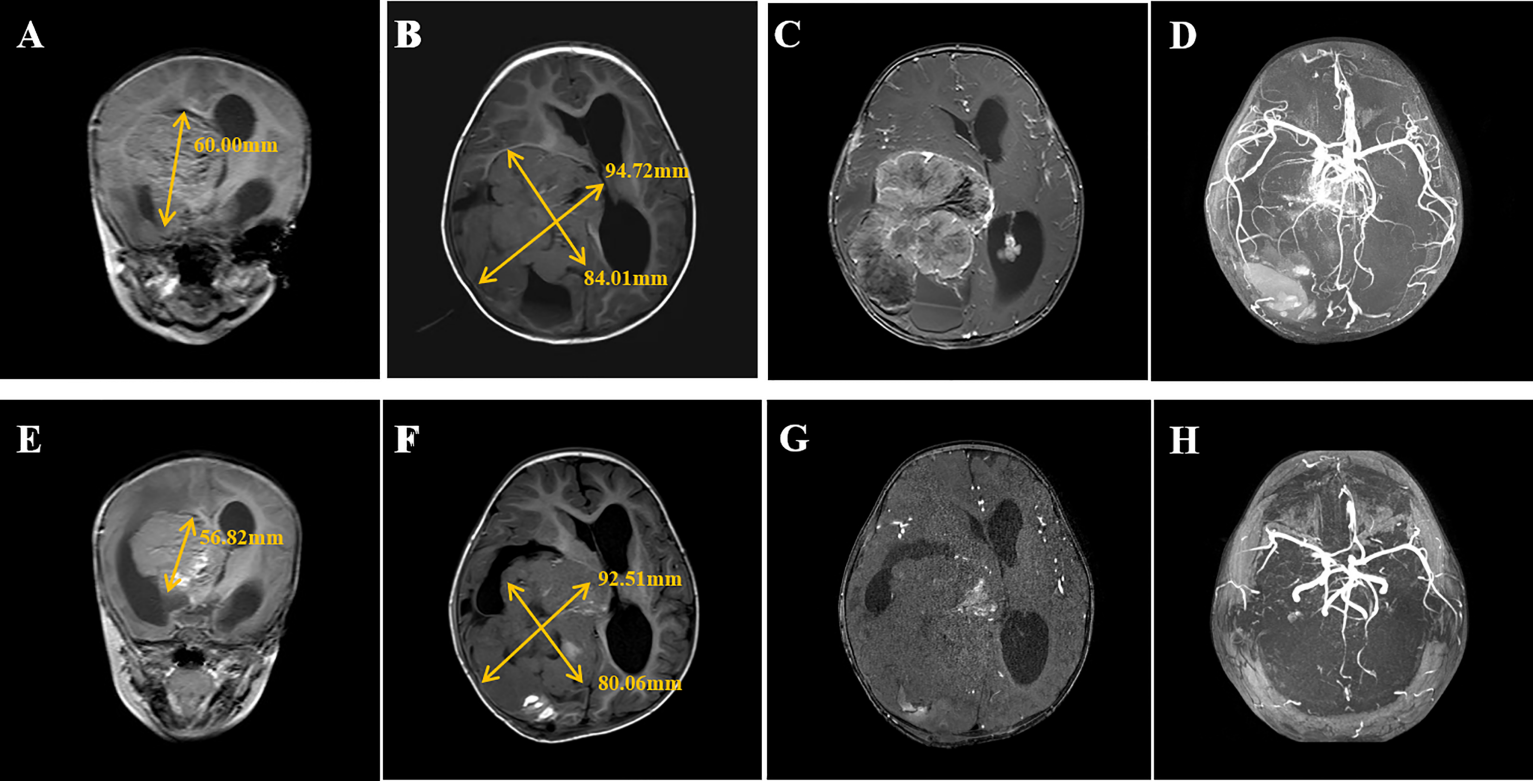
Serial intracranial MR images of the patient. Panel 1 Coronal **(A)** and transverse **(B)** T2-weighted MRI of the patient showed the tumor size in the right lateral ventricle and **(C, D)** MRA displayed the tumor with abundant blood supply (high-signal intensity) at admission (May 4, 2021). Panel 2 Coronal **(E)** and transverse **(F)** T2-weighted MRI showed a slight decrease in tumor size and **(G, H)** MRA displayed significant decrease of blood supply (high-signal intensity) to tumor after 14 days of oral rapamycin treatment (May 21, 2021).

To reduce blood supply to the tumor, the patient started taking 2.6 mg/m^2^/day rapamycin orally (divided into two equal doses) (2, 6, 7) on May 6. The patient had a normal diet (approximately 200 ml of formula milk every 3–4 h), and the 2 h postprandial plasma glucose of the patient ranged from 7.7 mmol/L to 10.8 mmol/L. The trough concentration of plasma rapamycin on May 10 after 4 days of rapamycin treatment was 6.8 ng/ml, below the target trough plasma rapamycin concentration of 10–15 ng/ml (6, 8), so we increased the dose of rapamycin to 5.2 mg/m^2^/day (divided into two equal doses). The patient became conscious with stable vital signs, and vomiting and neck resistance disappeared on May 13 after 7 days of rapamycin treatment (the 9th day after admission). Therefore, he underwent stereotactic biopsy to confirm tumor pathological diagnosis and Ommaya insertion to alleviate and prevent hydrocephalus (9, 10). The pathological diagnosis of the tumor in the biopsy tissue was aCPP. His fasting plasma glucose level rose to 21 mmol/L for no apparent reason on May 15 and returned to normal after 3 days of insulin injection. The trough concentration of plasma rapamycin on May 17 after 11 days of rapamycin treatment was 7.2 ng/ml, remaining below the target trough plasma rapamycin concentration of 10–15 ng/ml, so we continued to increase the dose of rapamycin to 7.8 mg/m^2^/day (divided into two equal doses) on May 19. The MR images showed that the blood supply to the tumor had notably decreased on May 21 after 14 days of rapamycin treatment (the 17th day after admission), while the volume of the tumor in the right lateral ventricle decreased to 56.82*80.06*92.51 mm (**Figure 2**). Thus, the patient successfully underwent a subtotal resection of the tumor (110*100*75 mm) on May 23 after 17 days of rapamycin treatment (the 19th day after admission), and the bleeding volume was less than 50 ml. However, after surgery, his recovery was complicated with pneumonia and intracranial staphylococcal infection, which was controlled with clindamycin (8 mg/kg, q8 h) and linezolid (10 mg/kg q12 h). He was also found to have hyperglycemia, rapamycin was withdrawn, and intravenous insulin therapy was given after excluding hyperglycemia caused by abnormal pancreatic structure and function. After 14 days of insulin treatment, his blood glucose concentration returned to normal, so he continued to take 7.8 mg/m^2^/day (divided into two equal doses) rapamycin orally on June 10 (the 16th day after rapamycin withdrawal), together with continuous subcutaneous insulin infusion (0.5 IU/h) to prevent hyperglycemia. The patient started receiving CPT-SIOP-2000 protocol chemotherapy (11, 12) on July 2. Because the tumor was well controlled, rapamycin and insulin were withdrawn on August 3 (**Figure 1**). At present, he is still undergoing chemotherapy and maintains normal plasma glucose and insulin levels, and the residual tumor continues to shrink.

## Discussion

CPTs with a rich blood supply have a fatal risk of hemorrhaging in the perioperative period and surgery (13–17), and a study found that CPT patients without preoperative vascular embolization lost 182% of their blood volume during surgery (18). In addition, the perioperative mortality of CPT patients can reach 25% (14, 15, 17, 19, 20), and hemorrhage accounts for 12% (20); therefore, it is necessary to reduce the tumor blood supply before surgery. Considering the risk of interventional therapy, the patient’s parents refused patient treatment with preoperative vascular embolization, so we hoped to reduce the blood supply to the tumor and improve the success rate of surgery by rapamycin treatment.

Rapamycin has been applied in brain tumors (4, 5, 21, 22) and complicated vascular anomalies (23, 24) in recent years. To reduce the blood supply to the tumor, the patient, in this case, took rapamycin preoperatively. The MR images showed that the blood supply to the tumor and tumor volume decreased after 17 days of rapamycin treatment, and the subtotal resection of the tumor was successful, with a bleeding volume of less than 50 ml (**Figure 2**).

The doses and courses of rapamycin vary based on the tumor type, location, and size. The recommended dose of rapamycin based on large-scale randomized scale trials of complicated vascular anomalies is 2.6 mg/m^2^/day (divided into two equal doses) (2, 6, 7) to achieve the target trough concentration of plasma rapamycin of 10–15 ng/ml (6, 8). However, there is no standard dose for treating solid tumors such as brain tumors. To reduce the blood supply to the tumor and tumor volume, the patient took 2.6 mg/m^2^/day rapamycin orally (divided into two equal doses) at the initial recommended dose of complicated vascular anomalies, but the trough concentration of plasma rapamycin was under 10–15 ng/ml after 7 days of rapamycin treatment. According to some reports, the recommended dose for children with brain tumors is 3–5 mg/m^2^/day (25–27), and the maximum dose for recurrent and refractory solid tumors reaches 150 mg/m^2^/day (once a week) without serious rapamycin-induced adverse effects (28). Furthermore, because of low oral bioavailability (15%–20%) (29) and poor blood–brain barrier penetration (30–32) [the ratio of the cerebrospinal fluid rapamycin concentration to the plasma rapamycin concentration was 0.0057 (33)], the patient with aCPP finally received 7.8 mg/m^2^/day (divided into two equal doses), and the trough concentration of plasma rapamycin reached 14.8 ng/ml. Of note, the trough concentration of plasma rapamycin reached 10–15 ng/ml when the dose of rapamycin was 2–3 times higher than the recommended dose, which was related to individual differences between patients.

The trough concentration of plasma rapamycin finally reached 10–15 ng/ml by adjusting the rapamycin dose in this case, but the patient developed hyperglycemia. After excluding hyperglycemia caused by pancreatic dysfunction, we suspected that the patient’s hyperglycemia was related to rapamycin treatment and that the concentration of plasma glucose was correlated with the rapamycin dose. Rapamycin-induced hyperglycemia could be controlled by insulin or dosage adjustment and returned to normal after rapamycin withdrawal, in this case, demonstrating that rapamycin-induced hyperglycemia was related to high-dose rapamycin. When the patient developed hyperglycemia, there were no other common adverse effects related to rapamycin treatment, such as oral ulcers, hyperlipidemia, liver dysfunction, and bone marrow suppression (34, 35), because the rapamycin concentration was within 15 ng/ml, and these common adverse effects were usually associated with concentrations of plasma rapamycin.

In conclusion, rapamycin can effectively reduce the blood supply to the tumor and tumor volume but induces reversible hyperglycemia when this aCPP patient received high-dose rapamycin orally to reach the target trough plasma concentration in this case. Therefore, the concentrations of plasma rapamycin and glucose should be monitored during high-dose rapamycin treatment, and dosage adjustment or insulin may be needed if patients develop hyperglycemia.

## Data Availability Statement

The raw data supporting the conclusions of this article will be made available by the authors, without undue reservation.

## Ethics Statement

Written informed consent was obtained from the minor(s)’ legal guardian/next of kin for the publication of any potentially identifiable images or data included in this article.

## Author Contributions

JL and ML drafted the manuscript. SL, ST, ZW, LYu, DL, LH, LW XLi JZ, XLa and YY collected materials and prepared figures. WZ and LYa critically revised the final manuscript. All authors contributed to the article and approved the submitted version.

## Conflict of Interest

The authors declare that the research was conducted without any commercial or financial relationships that could be construed as a potential conflict of interest.

## Publisher’s Note

All claims expressed in this article are solely those of the authors and do not necessarily represent those of their affiliated organizations, or those of the publisher, the editors and the reviewers. Any product that may be evaluated in this article, or claim that may be made by its manufacturer, is not guaranteed or endorsed by the publisher.
